# Gonococcal sepsis in a 32-year-old female: a case report

**DOI:** 10.1186/s13104-018-3346-1

**Published:** 2018-04-24

**Authors:** Michael Owusu, Kwadwo Sarfo Marfo, Godfred Acheampong, Abednego Arthur, Nimako Sarpong, Justin Im, Ondari D. Mogeni, Augustina Annan, Hsin-Ying Chiang, Chih-Horng Kuo, Se Eun Park, Florian Marks, Ellis Owusu-Dabo, Yaw Adu-Sarkodie

**Affiliations:** 1 Kumasi Centre for Collaborative Research in Tropical Medicine, Kwame Nkrumah University of Science and Technology, Kumasi, Ghana; 2 Department of Global Health, School of Public Health, Kwame Nkrumah University of Science and Technology, Kumasi, Ghana; 3 Agogo Presbyterian Hospital, Agogo, Ashanti Region Ghana; 4 Department of Theoretical and Applied Biology, Kwame Nkrumah University of Science and Technology, Kumasi, Ghana; 5 Institute of Plant and Microbial Biology, Academia Sinica, Taipei, Taiwan; 6 Department of Epidemiology, International Vaccine Institute, Seoul, South Korea; 7 Department of Clinical Microbiology, Kwame Nkrumah University of Science and Technology, Kumasi, Ghana

**Keywords:** *Neisseria gonorrhoeae*, Sepsis, Developing countries, Blood culture

## Abstract

**Background:**

*Neisseria gonorrhoeae* is a Gram-negative bacterium which affects the urethra, throat, rectum and cervix of patients and often associated with sexually transmitted infections. The global epidemiology of the disease is not well characterised especially in resource constraint countries due to poor diagnostic capacity and inefficient reporting systems. Although important, little is known about the propensity of this bacterium to cause sepsis in immunocompetent individuals.

**Case presentation:**

A 32-year-old female presented with fever and generalised malaise to a rural hospital in Ghana. The patient had previously been diagnosed as having enteric fever from a neighbouring health facility. Blood and urine samples were collected from the patient and cultured using standard microbiological and molecular techniques. *Neisseria gonorrhoeae* was isolated from the blood which was resistant to penicillin, ciprofloxacin and cotrimoxazole. The patient recovered following ceftriaxone and azithromycin treatment.

**Conclusion:**

This case highlights the importance of *N. gonorrhoeae* in causing sepsis and emphasises the need for blood culture investigation in diagnosis of patients presenting with fever.

## Background

*Neisseria gonorrhoeae* (*N. gonorrhoeae*) is a Gram-negative intracellular diplococcus bacterium associated with sexually transmitted infections (STIs). The organism causes gonorrhoeae; a disease which affects the urethra, throat, rectum and cervix of patients. The global epidemiology of the disease is not well characterised due to poor diagnostic capacity and inefficient reporting systems. Globally, it is estimated that about 106 million of occur annually [1]. It is the second commonly reported STI and is most prevalent in the age group of 15–49 years [2] Although about 87% of *N. gonorrhoeae* infections occur in symptomatic individuals, few infections (about 13%) progress asymptomatically [3]. The *N. gonorrhoeae* bacterium has become a major public health concern because of its increasing resistance to common antibiotics including penicillin, tetracycline, sulphonamides and, more recently, quinolones [1, 2].

In rare circumstances, gonococcal infection may result in sepsis and septic shock [4]. Gonococcal sepsis is most common in young women, but may develop in sexually active persons of any age. Reports on the occurrence of gonococcal sepsis in developing countries, especially within Africa are limited. Here, we report a case of gonococcal sepsis in a 32-year old female from a rural community of Ghana.

## Case presentation

A 32-year-old female trader presented to a hospital in the Ashanti region of Ghana, with 3-weeks history of generalised malaise and fever. Prior to her presentation, she was treated in a neighbouring hospital as a presumed case of enteric fever. On direct questioning, the patient had no known history of chronic underlying medical condition.

On examination, she weighed 73.2 kg, was slightly pale and febrile with a temperature of 38.5 °C. Examination of her body systems including the cervix and vagina were all normal. Based on the clinical findings, a provisional diagnosis of enteric fever was made.

Preliminary laboratory test for HIV and malaria were all negative. Her full blood count showed a low haemoglobin concentration of 8.7 g/dL and raised white cell count of 12.4 × 10^3^ cells/µL.

Liver function test showed high aspartate transaminase (AST) of 151 U/L and alanine transaminase (ALT) of 74 U/L. Her CD4 count was 1899 cells/mm^3^ and CD4/CD3 ratio was 0.71. Pending results for microbiological investigations, the patient was empirically administered with 500 mg of ciprofloxacin 12 hourly daily.

Blood and urine were collected for microbiological cultures. Blood sample was collected into Beckton Dickinson(BD) adult aerobic blood culture bottle (BD, Baltimore, US) and incubated in Bactec Machine at 35 °C (9050, BD).

The blood culture yielded a fastidious bacterium with small sized and creamy non-haemolytic colonies. Gram stain of the colonies showed Gram negative diplococci. Oxidase and catalase tests proved positive. The identity of the bacteria was determined by first using Analytic Profile Index (API) specific for *Neisseria* spp. and *Haemophilus* spp. (API NH, Biomerieux) and then confirming with 16S PCR method. The API kit showed a 100% consistency with *N. gonorrhoeae.*

For molecular identification, the 16S rRNA gene sequence of this bacterium was determined according to the procedure described previously [5]. Briefly DNA was extracted from the pure culture using Spherolyse extraction kit (Hain Lifesciense GmbH, Germany). The 16S rDNA was amplified using primer pair 8F and 1492R and the resulting sequence was checked using DECIPHER (version 2.2.0).The final sequence generated was deposited in NCBI GenBank under the *MF509590* and exhibits 99% (1440/1442) sequence identity to *N. gonorrhoeae* strain NCTC 8375 (NR_026079.1).

Antimicrobial susceptibility testing was performed on the isolate using the Kirby Baur disc diffusion method and following the Clinical and Laboratory Standards Institute guideline [6] The isolate showed resistance to ciprofloxacin, cotrimoxazole and penicillin but sensitivity to ceftriaxone, chloramphenicol and azithromycin. The patient’s prescription was amended to include ceftriaxone and azithromycin and her condition improved clinically.

## Discussions and conclusions

Gonococcal bacteremia is a rare condition affecting less than 3% of patients with gonorrhoea [7]. Isolated cases reported in the Korean population identified viral hepatitis and liver cirrhosis as risk factors for gonococcal bacteremia [8]. Other risk factors identified include the pathogenicity of the infecting strain, pregnancy, acquired complement deficiencies, systemic lupus erythematosus, sickle cell disease and splenectomy [8–10].

This patient does not have any of the underlying host factors mentioned above. Her HIV test was negative and a further CD4 cell population was also adequate thus ruling out any possibility of immunosuppression. We surmise that our patient might have been infected based on the virulence of the pathogen. It is possible that prolonged asymptomatic carriage of specific bacterial strains increases the risk of invasive diseases leading to sepsis. In the early 1980s, O’Brien determined that specific strains of *N. gonorrhoeae* with outer membrane porin isoform (PorB1b) seem to have increased permissive host cell invasion compared to others without those factors [9]. More studies on the pathogenicity and the genetic diversity of gonococcal strains would be helpful in understanding the mechanisms of gonococcal infections.

We also found the liver function markers (AST and ALT) of the patient as abnormal. Some authors have similarly reported abnormalities in liver enzymes of subjects [11, 12]. One common complication associated with gonococcal sepsis is perihepatitis [13]. Perihepatitis occurs by direct extension of the *N. gonorrhoeae* from the fallopian tubes to the liver capsule resulting in acute inflammation of the liver [13]. Other studies have similarly identified gonococcal bacteraemia in patients presenting with abnormal liver parameters, thus suggesting a possible association [11, 12].

Another important finding in this patient was the apparent lack of urogenital symptoms consistent with genital gonococcal infections. Thorough examination of the vagina and cervix did not show any signs of gonococcal infection. Other authors have similarly identified gonococcal infection in patients without history of urogenital symptoms [9]. About 50% of blood cultures were found to be positive in those cases although cultures from other body sites such as the synovial fluids and skin, were negative [14].

The isolated bacterium was also found to be resistant to common therapeutic agents including penicillin, ciprofloxacin and cotrimoxazole. This finding agrees with the recent report issued by the WHO on the growing gonococcal-associated antibiotic resistance and the need to strengthen antimicrobial surveillance of gonococcal infections [15].

Our report brings to fore the importance of taking blood cultures and administering the right kind of antibiotics for all fever related conditions that present to the hospital. This particular infection could have been missed if a blood culture had not been performed on the patient.

Further surveillance and investigation into the mechanisms of gonococcal invasion is therefore recommended infections.

## Abbreviations

STI: sexually transmitted infections
AST: aspartate transaminase
ALT: aspartate transaminase (AST)
BD: Beckton Dickenson
